# Dissecting Target Toxic Tissue and Tissue Specific Responses of Irinotecan in Rats Using Metabolomics Approach

**DOI:** 10.3389/fphar.2017.00122

**Published:** 2017-03-10

**Authors:** Yiran Yao, Pei Zhang, Jing Wang, Jiaqing Chen, Yong Wang, Yin Huang, Zunjian Zhang, Fengguo Xu

**Affiliations:** ^1^Key Laboratory of Drug Quality Control and Pharmacovigilance, Ministry of Education, China Pharmaceutical UniversityNanjing, China; ^2^Jiangsu Key Laboratory of Drug Screening, China Pharmaceutical UniversityNanjing, China; ^3^State Key Laboratory of Natural Medicine, China Pharmaceutical UniversityNanjing, China; ^4^School of Pharmacy, Shanxi University of Chinese MedicineXianyang, China; ^5^Jiangsu Institute for Food and Drug ControlNanjing, China

**Keywords:** CPT-11, metabolomics, jejunum, ileum, liver, tissue specificity

## Abstract

As an anticancer agent, irinotecan (CPT-11) has been widely applied in clinical, especially in the treatment of colorectal cancer. However, its clinical use has long been limited by the side effects and potential tissue toxicity. To discriminate the target toxic tissues and dissect the specific response of target tissues after CPT-11 administration in rats, untargeted metabolomic study was conducted. First, differential metabolites between CPT-11 treated group and control group in each tissue were screened out. Then, based on fold changes of these differential metabolites, principal component analysis and hierarchical cluster analysis were performed to visualize the degree and specificity of the influences of CPT-11 on the metabolic profiles of nine tissues. Using this step-wise method, ileum, jejunum, and liver were finally recognized as target toxic tissues. Furthermore, tissue specific responses of liver, ileum, and jejunum to CPT-11 were dissected and specific differential metabolites were screened out. Perturbations in Krebs cycle, amino acid, purine and bile acid metabolism were observed in target toxic tissues. In conclusion, our study put forward a new approach to dissect target toxic tissues and tissue specific responses of CPT-11 using metabolomics.

## Introduction

CPT-11 is an anticancer agent derived from camptothecin and has a wide anticancer spectrum including colorectal, pulmonary, cervical, and ovarian cancer (Mathijssen et al., 2001; Ikeguchi et al., 2011). *In vivo*, CPT-11 can be metabolized to its toxic form SN-38 through CE, and mainly distributed in liver, kidney, spleen, lung, and heart (Bardin et al., 2005; Hatfield et al., 2011). The wide distribution of CPT-11 and SN-38 in tissues enhanced the potential risk of tissue toxicity. Intestine was a well-recognized target toxic tissue of CPT-11. The administration of CPT-11 could cause goblet-cell hyperplasia, epithelial cell line disarrangement, and delayed diarrhea (Lima-Júnior et al., 2012; Spyropoulos, 2015). In addition, studies uncovered that CPT-11 has genetic influences on spleen and liver, and might cause steatohepatitis (Wang E. et al., 2011; McWhirter et al., 2013).

The clinical application of CPT-11 had been greatly limited by those side effects and potential tissue toxicity. However, there are no proper methods to rapidly and accurately identify the tissue toxicity. In toxicology studies, drug-induced tissue toxicity could be recognized by several techniques. Biochemical indicator analysis is the most routine one either in clinical or laboratory researches. However, the sensitivity of many indicators is much low. For example, in the evaluation of kidney function, blood urea nitrogen, and serum creatinine levels are frequently applied indexes. But only when glomerular filtration rate is lower than 50% will blood urea nitrogen and serum creatinine significantly elevate (Bonventre et al., 2010). On the other hand, some indicators have the problem of poor specificity. Glutamate pyruvic transaminase and aspartate transaminase are regularly used to evaluate liver function. However, other pathological conditions could also lead to the abnormal increases in glutamate pyruvic transaminase and aspartate transaminase (Korones et al., 2001). Histopathological examination is another common used means for the evaluation of drug-induced tissue toxicity. However, histological lesions often gradually come to the surface after a long drug administration period. Therefore, new strategies to sensitively, specially and rapidly recognize tissue damages induced by medicines are urgently needed.

Metabolomics is a branch of system biology that detects and quantifies the holistic metabolite compositions and their variations in integrated biological tissue systems (Bjerrum et al., 2010). Drug exposure might cause toxic effect on heart, spleen, lung, liver, and kidney etc., and the effects could be manifested as the alternation of tissue metabolic profile (Jiang et al., 2012). Our previous research has revealed the differential metabolites in serum between CPT-11 treated group and control group (Wang et al., 2015). In the present study we proposed a new metabolomics-based strategy, aiming to recognize the target toxic tissue and dissect the tissue specific responses of CPT-11 in rats.

## Materials and Methods

### Reagents and Chemicals

CPT-11 injection was obtained from Hengrui pharmaceuticals (Jiangsu, China). Methylamine hydrochloride, *N*-methyl-*N*-trifluoroacetamide, heptadecanoic acid, glibenclamide, pyridine, and standard compounds used for metabolite identification were purchased from Sigma–Aldrich (St. Louis, MO, USA). LC-MS grade reagents including methanol, acetonitrile and ethyl acetate were obtained from Honeywell (Burdick and Jackson, USA). Deionized water was purified using a Milli-Q system (Millipore, USA).

### Animal Experiment and Sample Collection

All animal experimental protocols were according to the guide for the care and use of laboratory animals (8th edition) released by the National Research Council of the National Academies, and all experimental protocol was approved by the Animal Ethics Committee of China Pharmaceutical University (License Number: SYXK 2012-0035).

Twenty-four male Sprague-Dawley rats (weighing 200 ± 20 g) were purchased from the Sino-British Sippr/BK Lab Animal Ltd (Shanghai, China) and housed in a temperature-controlled environment under 12/12 h dark/light cycle. Rats were acclimatized for a week with a standard rodent diet, and water was available *ad libitum*. Before drug administration, rats were randomly divided into two groups (control group and CPT-11-treated group, *n* = 12). At days 1 and 2, rats in CPT-11-treated group were administered with CPT-11 intravenously at the dosage of 150 mg/kg once a day for two consecutive days. Rats in control group received an equal volume of normal saline. Body weight and diarrhea score were monitored twice per day. At day 5, orbital venous blood samples were collected and prepared for blood test. After that, rats were euthanized and samples including jejunum, ileum, cecum, colon, liver, kidney, spleen, lung, and heart were removed. A portion of those tissues were fixed in 10% formalin for hematoxylin and eosin staining, and the remaining were stored at -80°C until metabolomics study. The strategy of dosing and sample collection time point was made according to our pre-experiments, our previous study, and existing literature (Trifan et al., 2002; Wang et al., 2015).

### Sample Preparation for Metabolomic Analysis

About 100 mg of each tissue were taken and added with precooled methanol (1:10, W/V). Then the mixtures were homogenized with a tissue homogenizer (Bioprep-24 homogenizer, China). The homogenate was then sonicated in an ice bath for 15 min. After centrifugation (16000 *g*, 10 min, 4°C) for twice, the supernatant were removed.

For GC-MS analysis, a 40 μL aliquot of the supernatant was added with 80 μL of methanol containing internal standard (heptadecanoic acid, 5 μg/mL). Thereafter the solution was vortex-mixed and then centrifuged at 16000 *g* for 10 min at 4°C. Then 80 μL of the supernatant was transferred to a brown glass vail and 25 μL *O*-methoxyamine hydrochloride (10 mg/mL in dry pyridine) was added. Subsequently, the solution was incubated at 37°C for 90 min and then evaporated to dryness at 50°C. Ultimately, 120 μL of *N*-methyl-*N*-trifluoroacetamide/ethyl acetate (1:1, V/V) was added. After incubation for 2 h at 37°C, the mixture was analyzed by GC-MS.

For LC-MS analysis, 40 μL aliquot was added with 80 μL of acetonitrile containing internal standard (glibenclamide, 5 μg/mL). The solution was vortex-mixed and then experienced two centrifugation at 16000 *g* for 10 min at 4°C. Finally, the supernatant was analyzed by LC-MS.

### Instrumental Analysis

Liquid chromatography-mass spectrometry analysis was performed on Shimadzu ultrafast LC-ion trap time-of flight MS system equipped with an electrospray ionization source (Shimadzu, Kyoto, Japan). Chromatographic separation was achieved on a Phenomenex Kinetex C_18_ column (100 mm × 2.1 mm, 2.6 μm, Phenomenex, USA). The gradient elution involved a mobile phase consisting of (A) 0.1% formic acid in water and (B) acetonitrile. The elution program was from 95% A to 5% A within 20 min and held for 3 min. The column oven was maintained at 40°C and the flow rate was 0.4 mL/min. The injection volume was 5 μL. The electrospray ionization-MS were acquired in both positive and negative ion mode with the interface voltage of 4.5 and -3.5 kV, respectively. The scan range was from mass to charge ratio 100 to 1000. The flow rate of nebulizing gas was 1.5 L/min and pressure of drying gas was 100 kPa. The temperature of heat block and curved desorption line were both 200°C. LCMS solution software (Shimadzu, Kyoto, Japan) was used for mass spectra acquisition and chromatograms processing.

Gas chromatography-mass spectrometry analysis was performed on Shimadzu GC/MS-QP2010 Ultra (Shimadzu, Kyoto, Japan) equipped with a 30.0 m × 0.25 mm (I.D) fused-silica capillary column with 0.25 μm Rtx-5MS stationary phase (Restek, USA). Helium was used as carrier gas and column flow was 1 mL/min. The injection volume was 1 μL and the split ratio was 50:1. The column temperature was initially kept at 70°C for 3 min and then increased at 10°C/min to 320°C, where it was held for 2 min. The injector temperature, interface temperature and ion source temperature were set at 250, 200, and 250°C, respectively. Ions were acquired in scan mode with mass to charge ratio from 45 to 600. Mass spectra and chromatograms were acquired and processed by GCMS solution software (Shimadzu, Kyoto, Japan).

### Data Quality Assurance

Quality control samples were prepared by pooling equal aliquot of each tissue homogenate and treated congruously with real samples. In order to monitor the robustness of sample preparation and the stability of instrument analysis, QC samples were intermittently injected through the analytical experiment. During the instrument analysis, all samples from control and CPT-11-treated group were randomized in order to avoid inter-batch differences. Unsupervised pattern recognition method PCA was constructed based on QC samples and real samples to evaluate the data quality.

### Data Preprocessing and Analysis

Each chromatogram obtained was processed by profiling solution (Version 1.1, Shimadzu, Japan) for peak deconvolution and alignment. The primary parameters were set referring to our previous study (Wang et al., 2015). The resulting data were exported to an Excel table (Microsoft, USA), handled according to the “80%” rule: only the variables with values above zero in at least 80% of one or more groups were kept. Then, the individual ion fragment intensity was normalized to the sum intensity of all peaks in one chromatogram. After normalization, variables with relative standard deviation lower than 30% in QC samples were kept for further data analysis.

The preprocessed data sets were imported into SIMCA-P software (Version 13.0, Umetrics, Sweden). Before the performance of PCA and OPLS-DA, variables were scaled by Pareto scaling method (van den Berg et al., 2006). In OPLS-DA, variables with variable importance projection value exceeding 1.0 were screened out for being responsible for the differences between CPT-11-treated group and control group in OPLS-DA. Furthermore, Mann–Whitney *U* test was performed to determine the significance of each variable using Multi-experiment Viewer (MeV^1^).

### Potential Biomarkers Identification and Visualization

Preliminary identification of metabolites detected by GC-MS was performed with GCMSsolution by comparing with embedded National Institute of Standards and Technology library. Peaks with similarity percent more than 70% were assigned for compound names referring to existing literatures. Metabolites detected in LC-MS were firstly identified by comparing accurate mass to charge ratio, retention time and MS/MS fragmentation patterns with those standard compounds available in our lab. Other metabolites without standards were identified by searching online databases like HMDB^2^ and METIN^3^ etc. Heat map was used to visualize the change trends between groups in each tissue and drawn by using Multi-experiment Viewer (MeV^4^).

### Target Toxic Tissue Dissecting by HCA and PCA

**FIGURE 1:**
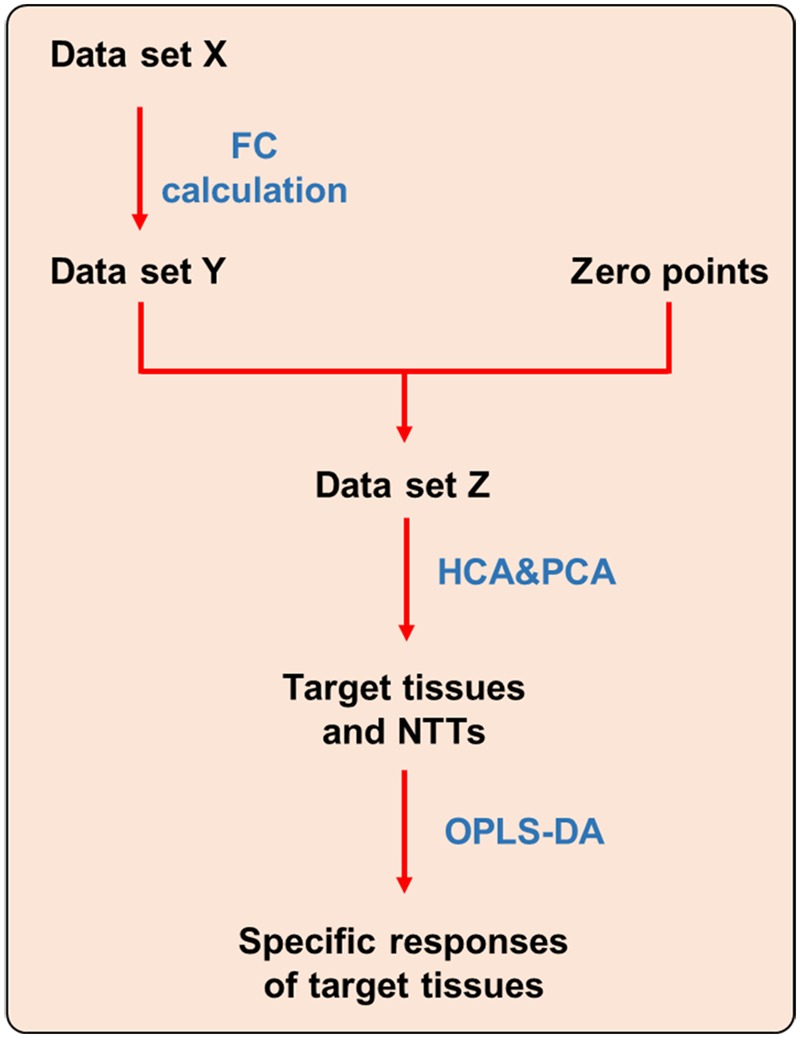
**Strategy for dissecting target tissue and tissue specific response.** NTT: non-target tissue.

Differential metabolites screened out from each tissue were combined into a new metabolite collection named Data set X (see **Figure 1**). Fold change (FC) of each metabolite were calculated using the following formulas. FCs of metabolites that were not screened out in certain tissue were replaced with 0.

$$\text{if}\;{\text{M}}_{\text{n}}\;<\;{\text{C}}_{\text{ave}},\;\text{then}\;\text{FC}\;=\;({\text{M}}_{\text{n}}{\text{-C}}_{\text{ave}}){\text{/M}}_{\text{n}}$$

$$\text{if}\;{\text{M}}_{\text{n}}\;>\;{\text{C}}_{\text{ave}},\;\text{then}\;\text{FC}\;=\;({\text{M}}_{\text{n}}{\text{-C}}_{\text{ave}}){\text{/C}}_{\text{ave}}$$

where M_n_ represents the value of one metabolite in one tissue in Data set X, and C_ave_ represent the average value of this metabolite in control group of this tissue.

After FC calculation, the new data set was defined as Data set Y. In order to make the data obtained from different tissues be comparable, zero points were set using the following stepwise method. Firstly, the same volume of each tissue homogenate were mixed up and randomly divided into two sub-groups (S1 and S2) with six samples in each group. Second, the samples in S1 and S2 group were processed and analyzed using the same method as other tissues, and only those metabolites covered by Data set Y were picked out. Third, FC of each metabolite in S2 group was calculated by dividing with the corresponding mean value in S1 group. Those FCs were defined as zero points. Finally, a new data set was generated by combining zero points with all tissues named as Data set *Z*, which was then imported to SPSS and SIMCA-P software to perform HCA and PCA, respectively. In HCA, tissues with larger distances were thought as most affected by CPT-11. And in PCA, the tissues had farther distances from zero points were recognized as the target toxic tissues of CPT-11.

### Tissue Specific Response Revealed by OPLS-DA

In order to reveal the tissue specific response of target tissues, OPLS-DA was performed to discriminate target tissues from the rest NTT. Differential metabolites were screened out to represent the specific responses of each target tissue to CPT-11 exposure (**Figure 1**).

## Results

### Blood Test Results

**FIGURE 2:**
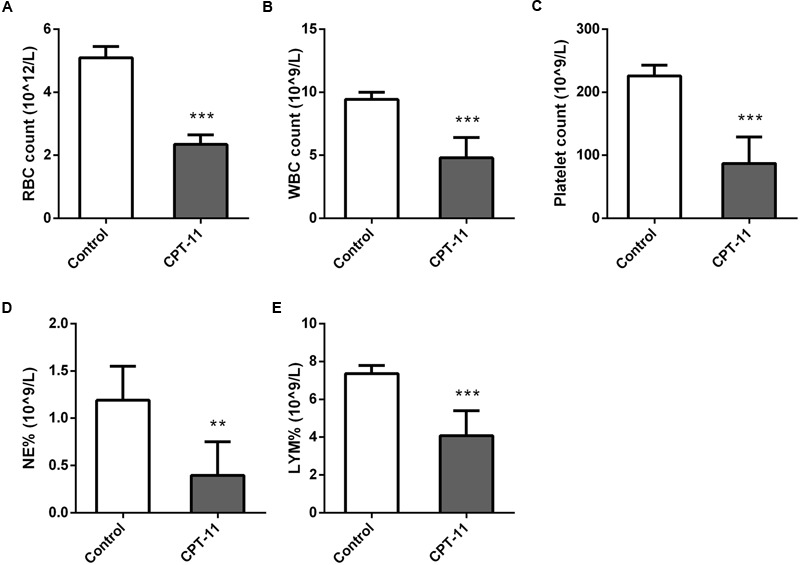
**Column plots of blood test results. (A)** RBC count; **(B)** WBC count; **(C)** Platelet count; **(D)** NEUT count; **(E)** LYMH count. Mann–Whitney *U* test, ^∗∗^*p* < 0.01, ^∗∗∗^*p* < 0.001, in comparison with control group.

Red blood cell (RBC) count, white blood cell (WBC) count, platelet count, neutrophil (NEUT) count and lymphocyte (LYMH) count results were shown in **Figure 2**. There were all significant differences between control and CPT-11 treated groups.

### Pathological Analysis

**FIGURE 3:**
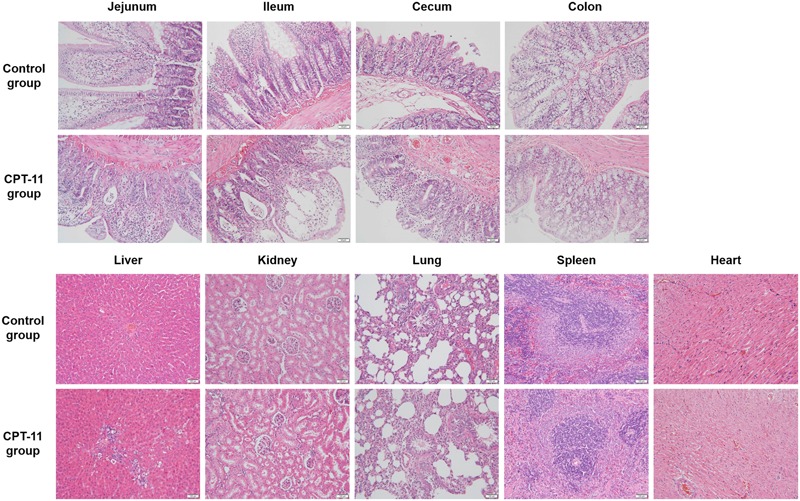
**Hematoxylin and eosin staining of four sections of intestine and other tissues including kidney, liver, spleen, lung, and heart**.

As can be seen in **Figure 3**, pathological changes were observed in intestines, liver and kidney. All intestinal tissues were injured and manifested as epithelial cells degeneration, intestinal villus shortening and glandular lumen expansion. Ileum was the most damaged among the four intestinal segments. In liver, mild partial inflammatory cells infiltration, cell spotty necrosis, eosinophilic body appearance and vacuolar degeneration were observed. In kidney, there was mild hydropic degeneration of renal tubular epithelial cells. No lesion was observed in the rest tissues.

### Data Quality Assurance

Data quality was evaluated using QC samples. For data acquired from both GC-MS and LC-MS, QCs in PCA score plot clustered well regarding for all the nine tissues, indicating good reproducibility of the sample process procedure and the instrumental system.

### Metabolic Profiling of Tissues Were Disturbed by CPT-11

**FIGURE 4:**
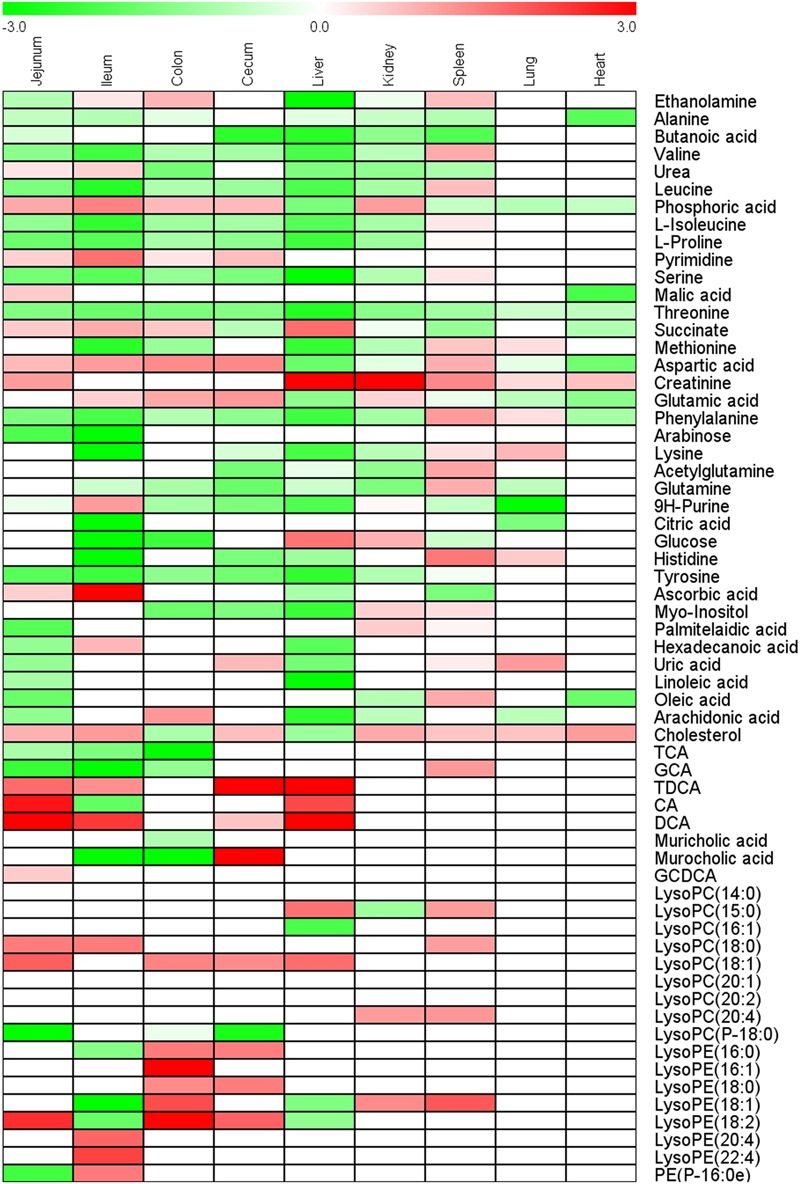
**Heat map of fold-change of metabolites screened out in ileum, jejunum, cecum, colon, heart, lung, liver, kidney, and spleen**.

Principal component analysis and OPLS-DA models of all nine tissues were constructed between control group and CPT-11-treated group (Supplementary Figure S1). Intestines of rats including ileum, cecum, colon, and jejunum in CPT-11-treated group were completely separated from control group in PCA score plots, suggesting obvious metabolic changes in intestines were induced due to CPT-11 administration. Finally, 29, 37, 28, and 28 differential metabolites were screened out in jejunum, ileum, cecum, and colon, respectively. In addition to intestines, liver samples of CPT-11-treated group were also greatly separated with control group in PCA score plot. Ultimately, 32 significantly changed metabolites were screened out in liver. Only 11 metabolites were found significantly changed in heart, indicating less influence was induced by CPT-11. Differential metabolites with change trend between control group and CPT-11-treated group of different tissues were listed in Supplementary Table S1. Heat map was utilized to express content difference visually and the results were shown in **Figure 4**.

### Multivariate Statistical Analysis Recognized Ileum, Jejunum, and Liver as Toxic Target Tissues

**FIGURE 5:**
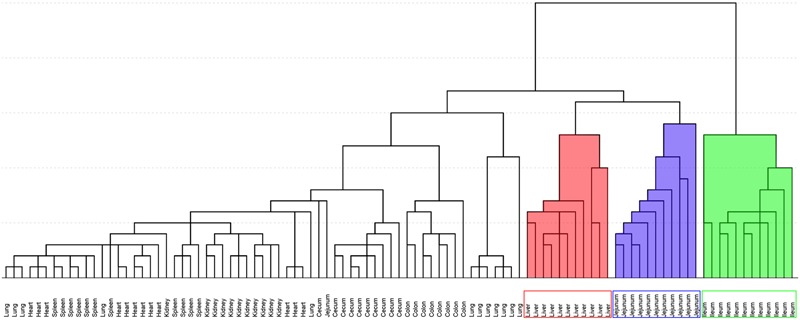
**Hierarchical cluster analysis of fold-change of metabolites screened out in nine tissues.** Background filled in red, blue and green were liver, jejunum, and ileum samples, respectively.

**FIGURE 6:**
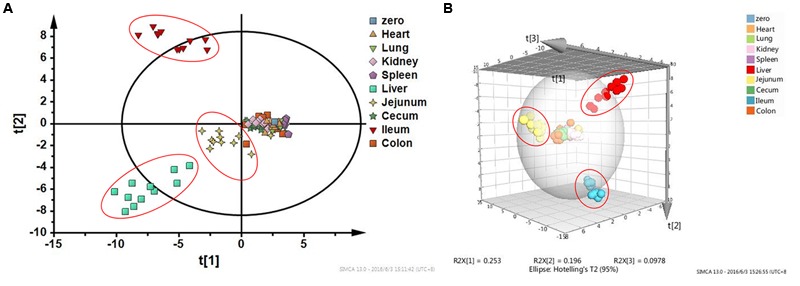
**Principal component analysis score plots based on fold-change data of metabolites screened out in nine tissues. (A)** Two-dimensional PCA score plot; **(B)** Three-dimensional PCA score plot. Liver, jejunum, and ileum samples were highlighted with red circles.

**Figure 5** shows the result of HCA. Distance of two groups represented the similarity of metabolic changes in each tissue. Ileum, jejunum, and liver had farther distances from the other tissues. Besides, PCA score plot (**Figure 6**) showed the similar results with HCA. As can be seen from the 2-dimentional (**Figure 6A**) and 3-dimentional score plots (**Figure 6B**), ileum, liver, and jejunum were completely distinguished from other tissues and had farther distances from zero points than all the other tissues. HCA and PCA results indicated that CPT-11 could cause specific influences on the metabolic profiles of ileum, liver, and jejunum. Other tissues were less affected.

### Dissecting Tissue Specific Responses to CPT-11 Using OPLS-DA

Ileum, jejunum, and liver were recognized as toxic target tissues according to the results of pathological study and multivariate statistical analysis. For dissecting specific toxic responses to CPT-11, OPLS-DA was used to screen differential metabolites that could distinguish toxic target tissues from NTTs (tissues except liver, ileum, and jejunum).

**FIGURE 7:**
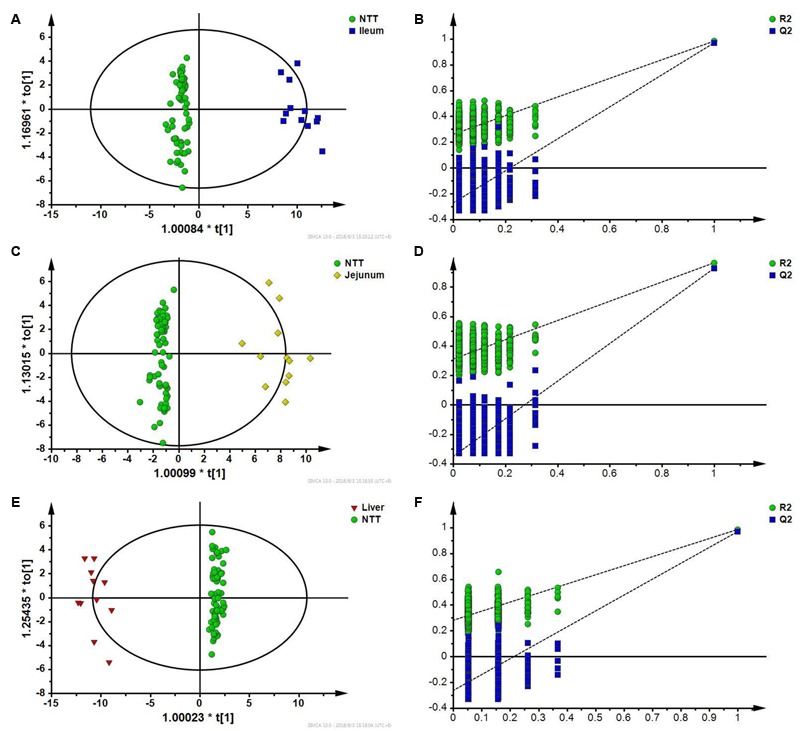
**(A)** OPLS-DA score plot of ileum and NTTs; **(B)** Permutation test of OPLS-DA model constructed between ileum and NTTs; **(C)** OPLS-DA score plot of jejunum and NTTs; **(D)** Permutation test of OPLS-DA model constructed between jejunum and NTTs; **(E)** OPLS-DA score plot of liver and NTTs; **(F)** Permutation test of OPLS-DA model constructed between liver and NTTs.

First, specific responses of ileum to CPT-11 were analyzed by comparing ileum with NTTs. The two groups could be well separated with the model parameter *Q*^2^ of 0.977 in OPLS-DA score plot (**Figure 7A**). Permutation test showed that the model was not overfitting (**Figure 7B**). It is indicated that the change of metabolites in ileum can be distinguished from NTTs. After variable screening and metabolite identification, bile acids including cholic acid, deoxycholic acid, taurocholic acid, taurodeoxycholic acid, and glycocholic acid, pyrimidine, lyso-phosphatidylethanolamines, metabolites related to energy metabolism like glucose, citric acid and succinic acid, amino acids including isoleucine, methionine, histidine, leucine, lysine, serine, valine, tyrosine, phenylalanine, proline, and threonine, and ascorbic acid were screened out. Pathway analysis revealed that the intestinal related toxic responses involved bile acid, energy and amino acid metabolism.

Jejunum was also distinguished from NTTs according to OPLS-DA results (**Figure 7C**), and permutation test showed that the model was not overfitting (**Figure 7D**). Differential metabolites were bile acids like cholic acid and deoxycholic acid, lyso-phosphatidylethanolamines, linoleic acid, palmitic acid, and palmitelaidic acid, amino acids including isoleucine, leucine, proline, tyrosine, serine, valine, and phenylalanine, and uric acid.

In OPLS-DA score plot (**Figure 7E**), metabolic profile of liver could be distinguished from NTTs. Permutation test also showed that the model was not overfitting (**Figure 7F**). Amino acids including threonine, methionine, proline, serine, valine, isoleucine, leucine, lysine, tyrosine, phenylalanine, glutamic acid, and histidine, free fatty acids including palmitic acid, linoleic acid and arachidonic acid, taurodeoxycholic acid, succinic acid, and cholesterol were finally identified as specific responses of liver.

## Discussion

### Target Toxic Tissues of CPT-11

In the present study, ileum, jejunum, and liver were recognized as target toxic tissues of CPT-11 in rats. The findings were in accordance with previous reports that ileum was the most damaged among all intestinal segments in CPT-11 treated mice (Nakao et al., 2012). CPT-11 is partly transformed into SN-38 in liver by CEs, and further transformed into SN-38G by UDP glucuronosyl transferase1A1 in liver. SN-38G is excreted to ileum together with bile and transformed into SN-38 by β-glucuronidase produced by gut flora (Takasuna et al., 1996), which can be toxic to intestinal epithelial cell (Gupta et al., 1994; Mathijssen et al., 2001). Jejunum was also reported to be damaged by CPT-11 in some researches since it has large distribution of CEs which can be an activator for the transformation of CPT-11 to SN-38 (Guichard et al., 1999; Lam et al., 2010). Liver is the most vital organ for the metabolism and detoxification of CPT-11 (Humerickhouse et al., 2000). It has reported that a long period of CPT-11 administration could cause steatohepatitis, mechanisms of which were mitochondrial impairment and inflammation induced by impaired β-oxidation (Costa et al., 2014). Therefore, it is not surprising that metabolic profile of liver was greatly influenced.

### Specific Responses of Target Toxic Tissues

Specific responses of ileum were identified in the present study. Bile acid metabolism is related to hepatocyte and intestinal bacteria (Danielsson and Sjovall, 1975), which can be influenced by intestinal flora disturbance and liver disease. It was reported that bile acid metabolism can be disturbed in inflammatory bowel diseases and had close relationship with inflammatory reaction (Vavassori et al., 2009; Lenicek et al., 2011). Ascorbic acid is an antioxidant with the capability of rapidly scavenging a number of reactive oxygen species (Buffinton and Doe, 1995). Ascorbic acid was in greater quantities in intestine during catabolic conditions and corresponding high loads of oxidative stress, which was found in the inflammatory process of ulcerative colitis (Wang et al., 2008).

Similarly, some specific responses of jejunum to CPT-11 were recognized. Bile acids in humans are synthesized from cholesterol in the liver, and conjugated to either glycine or taurine. Bile salts are secreted into the small intestine as a component of bile and transformed into deoxycholic acid by gut flora (Ridlon et al., 2006). CPT-11 was reported to alter intestinal content of deoxycholic acid which potentiated the suppression of IL-10 and enhanced the intestinal damage (Lin et al., 2012). Dietary palmitic acid was reported to be able to modulate intestinal re-growth after massive small bowel resection in rats. It was reported that linoleic acid could accelerate mucosa regeneration after small bowel resection (Park et al., 1989). The decreased amount of linoleic acid might have influences on the repair course of damaged jejunum.

Also, specific responses in liver were discriminated. Taurodeoxycholic acid was found to be increased in liver in the present study, which was consistent with the change trend in another research about metabolic profiling of bile acids in livers of CPT-11 treated rats (Fang et al., 2016). Taurodeoxycholic acid is formed in the liver by conjugation of deoxycholic acid with taurine and reported to be increased in non-alcoholic steatohepatitis livers (Lake et al., 2013). Methionine is a precursor to phosphatidylcholine, the main phospholipid coating very low-density lipoprotein particles which carry triglycerides and cholesterol into blood (Vance and Vance, 1985). Methionine was also proved to stimulate the secretion of very low-density lipoprotein from cultured rat hepatocytes under the condition of choline deficiency (Yao and Vance, 1988). The decrease of methionine could also have an effect on the synthesis of *S*-adenosylmethionine which is related to hepatic oxidative stress and lipid peroxidation damage (Rinella et al., 2008). Energy metabolism was enhanced with up-regulated succinic acid and malic acid, which may be related with the dysfunction of mitochondrion. Palmitic acid was proved to be beneficial to alcoholic fatty liver due to the down-regulation of lipid peroxidation (Nanji et al., 1995). In our research, palmitic acid was decreased specially in liver tissue which is noxious to liver health especially in abnormal conditions. Linoleic acid was decreased in liver tissue in this study and was also reported to be decreased in livers of rats with non-alcohol fatty liver disease (Wang X. et al., 2011). Linoleic acid is an important polyunsaturated fatty acid which was reported to be able to alleviate the steatosis in livers of mice with non-alcohol fatty liver disease. n-3 polyunsaturated fatty acid could activate PPAR-α to up-regulate the expression of gene which participates in fatty acid oxidation in liver (Videla et al., 2004). Polyunsaturated fatty acid was also reported to increase the secretion of ApoB-100 to enhance the transport of triglyceride (Buang et al., 2004). Ethanolamine is the second most abundant head group for phospholipids existed on cell membranes and the component of palmitoylethanolamide used as messenger molecules (Yeagle, 1989; Verme et al., 2005). The decrease of ethanolamine could interfere the intercellular message transmission.

## Conclusion

Metabolomics combined with multivariate statistical analysis method was performed to recognize the toxic target tissues of CPT-11 in rats. Ultimately, liver, ileum, and jejunum was distinguished as the most affected tissues. Specific metabolites changed in these target tissues were screened out by OPLS-DA. It is found that the perturbations of Krebs cycle, amino acid metabolism, purine metabolism and bile acid metabolism were occurred in toxic tissues. This work demonstrates that metabolomics approach is a potentially powerful tool for recognizing toxic target tissues and can be utilized to dissect specific biochemical responses of target tissues. It is believed that the work flow of dissecting target toxic tissue based on metabolomics and multivariate statistical analysis could be extended to other drug tissue toxicity researches.

## Author Contributions

YY performed the majority of the experiment. YY and PZ wrote and revised the manuscript. JW, JC, YW, and YH supported several experiments. FX and ZZ supervised the research and revised the manuscript.

## Conflict of Interest Statement

The authors declare that the research was conducted in the absence of any commercial or financial relationships that could be construed as a potential conflict of interest.

## Footnotes

1 http://www.tm4.org/

2 http://www.hmdb.ca/

3 http://metlin.scripps.edu/

4 http://www.tm4.org/

## Abbreviations

CE: carboxylesterase
GC-MS: gas chromatography-mass spectrometry
HCA: hierarchical cluster analysis
LC-MS: liquid chromatography-mass spectrometry
NTT: non-target tissue
OPLS-DA: orthogonal partial least squares-discriminant analysis
PCA: principle component analysis
QC: quality control
SN-38: 7-ethyl-10-hydroxycamptothecin
SN-38G: SN-38 glucuronide
