# Supplementary material for: Dissecting Target Toxic Tissue and Tissue Specific Responses of Irinotecan in Rats Using Metabolomics Approach
Source: Front Pharmacol. 2017 Mar 10;8:122. doi: 10.3389/fphar.2017.00122 (PMC5344918; doi:10.3389/fphar.2017.00122)
Supplement: Supplementary file 1 [file Presentation_1.pdf]

Supplementary Material

Dissecting target toxic tissue and tissue specific responses of irinotecan in rats using metabolomics approach

Yiran Yao[1,2,3#], Pei Zhang[1,2,3#], Jing Wang[4], Jiaqing Chen[1,2,3], Yong Wang[5], Yin Huang [1,2,3], Zunjian Zhang [1,2,3,*], Fengguo Xu [1,2,3,*]

[1] Key Laboratory of Drug Quality Control and Pharmacovigilance (Ministry of Education), China Pharmaceutical University, Nanjing 210009, China

[2] Jiangsu Key Laboratory of Drug Screening, China Pharmaceutical University, Nanjing 210009, China

[3] State Key Laboratory of Natural Medicine, China Pharmaceutical University, Nanjing 210009, China

[4] School of Pharmacy, Shanxi University of Chinese Medicine, Xianyang 712046, China

[5] Jiangsu Institute for Food and Drug Control, Nanjing 210008, China

# these authors have contributed equally to this paper

Corresponding authors:

[*] Prof. Fengguo Xu

E-mail: fengguoxu@gmail.com

[*] Prof. Zunjian Zhang

E-mail: zunjianzhangcpu@hotmail.com

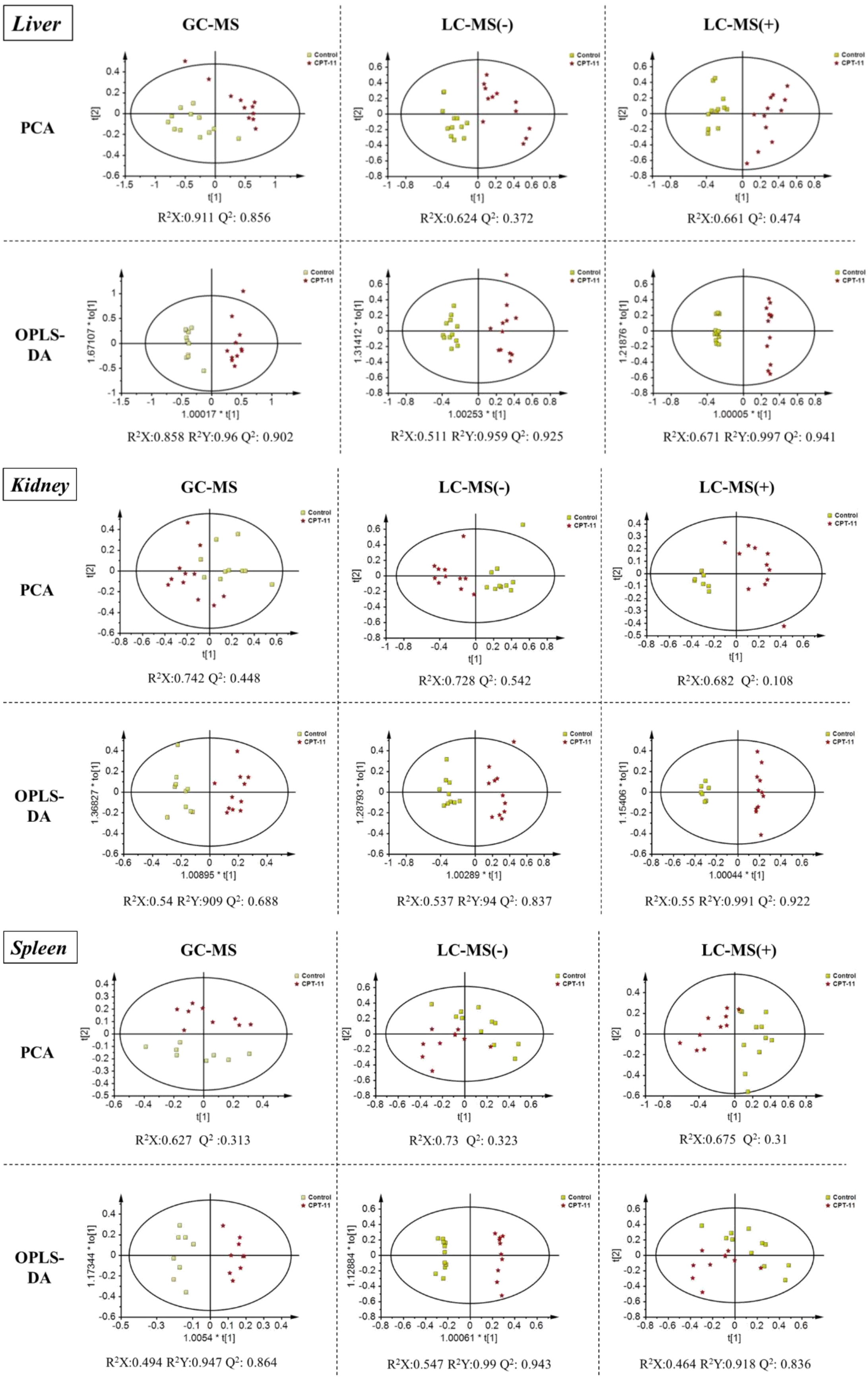
Liver
GC-MS
LC-MS(-)
LC-MS(+)
PCA
R²X:0.911 Q²: 0.856
R²X:0.624 Q²: 0.372
R²X:0.661 Q²: 0.474
OPLS-DA
R²X:0.858 R²Y:0.96 Q²: 0.902
R²X:0.511 R²Y:0.959 Q²: 0.925
R²X:0.671 R²Y:0.997 Q²: 0.941
Kidney
GC-MS
LC-MS(-)
LC-MS(+)
PCA
R²X:0.742 Q²: 0.448
R²X:0.728 Q²: 0.542
R²X:0.682 Q²: 0.108
OPLS-DA
R²X:0.54 R²Y:909 Q²: 0.688
R²X:0.537 R²Y:94 Q²: 0.837
R²X:0.55 R²Y:0.991 Q²: 0.922
Spleen
GC-MS
LC-MS(-)
LC-MS(+)
PCA
R²X:0.627 Q² :0.313
R²X:0.73 Q²: 0.323
R²X:0.675 Q²: 0.31
OPLS-DA
R²X:0.494 R²Y:0.947 Q²: 0.864
R²X:0.547 R²Y:0.99 Q²: 0.943
R²X:0.464 R²Y:0.918 Q²: 0.836
Control
CPT-11

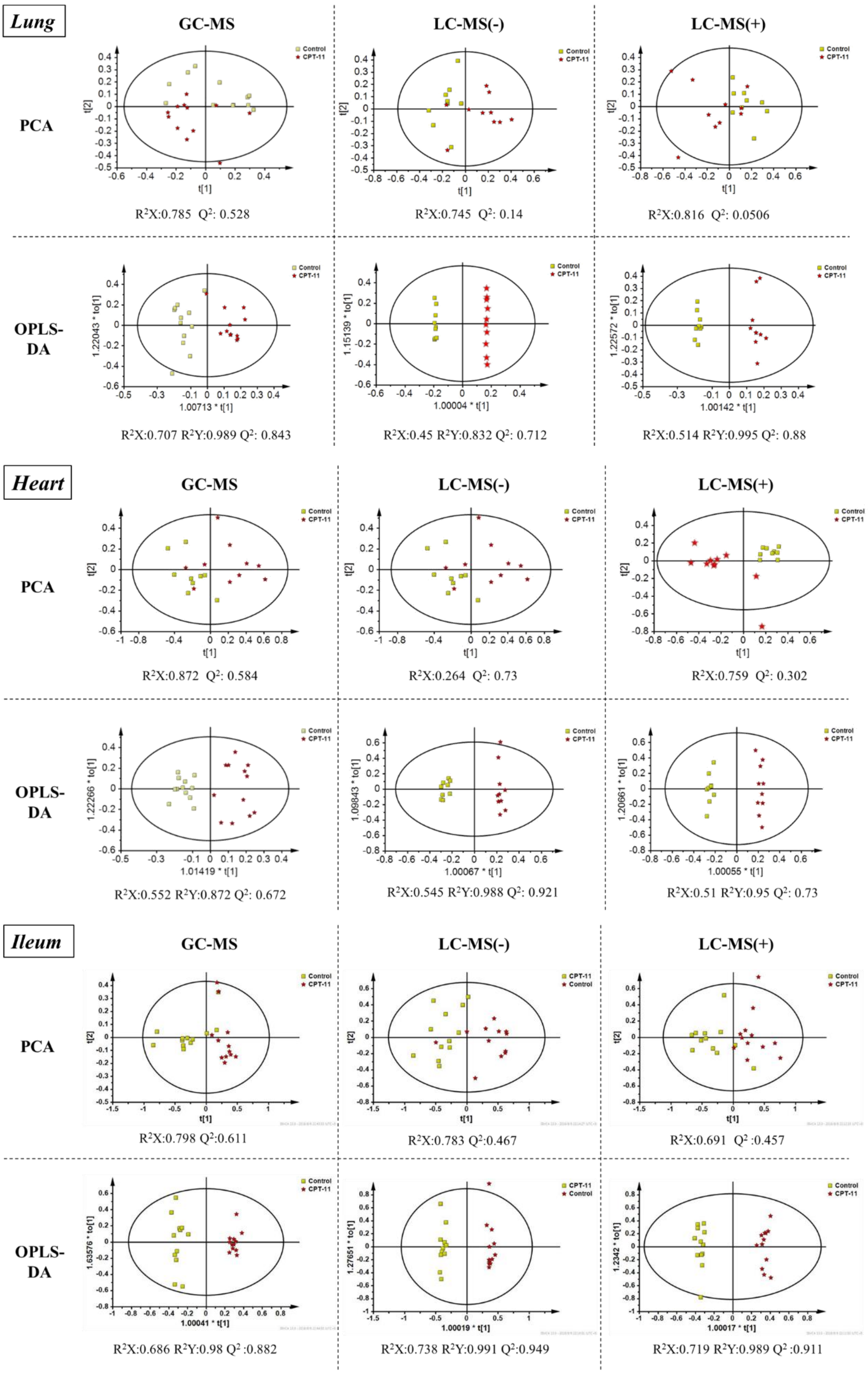
Lung
GC-MS
LC-MS(-)
LC-MS(+)
PCA
R²X:0.785 Q²: 0.528
R²X:0.745 Q²: 0.14
R²X:0.816 Q²: 0.0506
OPLS-DA
R²X:0.707 R²Y:0.989 Q²: 0.843
R²X:0.45 R²Y:0.832 Q²: 0.712
R²X:0.514 R²Y:0.995 Q²: 0.88
Heart
GC-MS
LC-MS(-)
LC-MS(+)
PCA
R²X:0.872 Q²: 0.584
R²X:0.264 Q²: 0.73
R²X:0.759 Q²: 0.302
OPLS-DA
R²X:0.552 R²Y:0.872 Q²: 0.672
R²X:0.545 R²Y:0.988 Q²: 0.921
R²X:0.51 R²Y:0.95 Q²: 0.73
Ileum
GC-MS
LC-MS(-)
LC-MS(+)
PCA
R²X:0.798 Q²:0.611
R²X:0.783 Q²:0.467
R²X:0.691 Q²:0.457
OPLS-DA
R²X:0.686 R²Y:0.98 Q²:0.882
R²X:0.738 R²Y:0.991 Q²:0.949
R²X:0.719 R²Y:0.989 Q²:0.911
Control
CPT-11
t[1]
t[2]

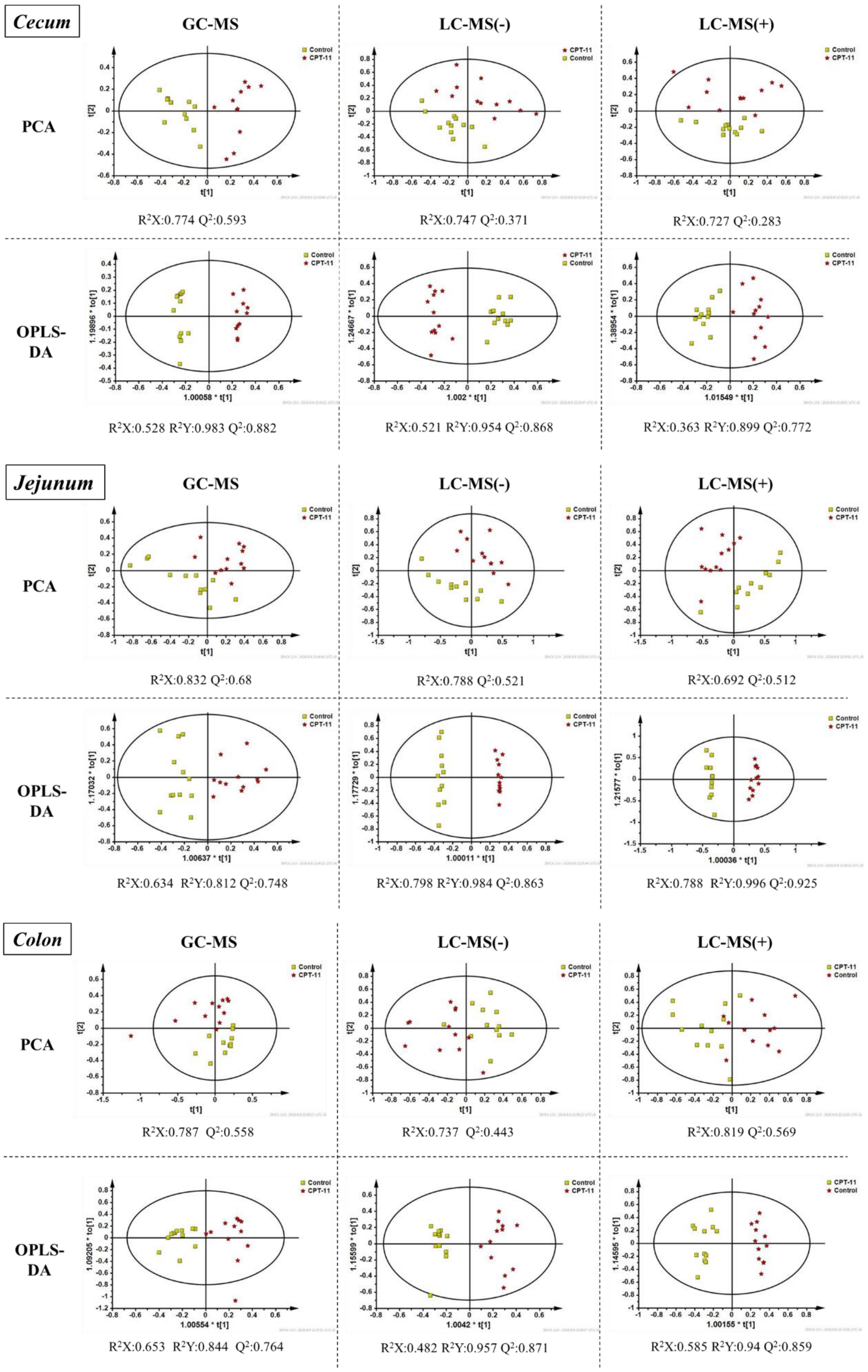

Figure S1 PCA and OPLS-DA score plots of different tissues between control group and CPT-11 treated group.

Table S1 Change trends of differential metabolites in different tissues in CPT-11 treated group.

Metabolites	Jejunum	Ileum	Cecum	Colon	Liver	Kidney	Spleen	Lung	Heart
Ethanolamine	-	-	-	↑*	↓*	-	↑*	-	-
Alanine#	↓*	↓*	-	-	-	↓*	↓*	-	↓*
Butanoic acid	-	-	↓*	-	↓*	-	↓*	-	-
Valine#	↓*	↓*	↓*	↓*	↓*	↓*	↑*	↓*	-
Urea#	-	↑*	-	↓*	↓*	↓*	-	↓*	-
Leucine#	↓*	↓*	↓*	↓*	↓*	↓*	↑*	↓*	-
Phosphoric acid	↑*	↑*	↑*	↑*	↓*	↑*	-	↓*	↑*
Isoleucine#	↓*	↓*	↓*	↓*	↓*	↓*	↑*	↑*	-
Proline#	↓*	↓*	↓*	↓*	↓*	↓*	-	↑*	-
Pyrimidine	-	↑*	↑*	-	-	-	-	-	-
Serine#	↓*	↓*	↓*	↓*	↓*	↓*	-	-	-
Threonine#	↓*	↓*	↓*	↓*	↓*	↓*	↓*	↓*	↓*
Succinic acid#	-	-	↓*	↑*	↑*	↓*	↓*	↑*	↓*
Methionine	-	↓*	-	↓*	↓*	↓*	-	-	-
Aspartic acid#	↑*	↑*	↑*	↑*	↓*	-	↑*	↑*	↓*
Creatinine#	↑*	-	-	-	↑*	↑*	-	-	-
Glutamic acid#	-	↑*	↑*	↑*	↓*	↓*	-	-	↓*
Phenylalanine#	↓*	↓*	↓*	-	↓*	-	↑*	↑*	-
Lysine#	-	↓*	-	-	↓*	-	-	↑*	-
4-hydroxy-proline	-	-	-	-	-	-	-	↓*	-
N-Acetylglutamine	-	-	↓*	-	-	-	-	-	-
Glutamine#	-	-	↓*	↓*	↓*	↓*	-	-	-
9H-Purine	-	↑*	↓*	↓*	↓*	-	-	↓*	-
Citric acid#	-	↓*	-	-	-	-	-	↓*	-
Glucose	-	↓*	-	↓*	↑*	↑*	-	↓*	↑*
Histidine	-	↓*	↓*	-	↓*	-	↑*	↑*	-
Tyrosine#	↓*	↓*	↓*	↓*	↓*	↓*	-	↓*	-
Myo-Inositol	-	-	↓*	↓*	-	-	↓*	-	-
Palmitic acid	↓*	↑*	-	-	-	-	-	↓*	↓*
Uric acid	↓*	-	-	-	-	-	-	↓*	-
Linoleic acid#	↓*	-	-	-	↓*	-	-	-	-
Oleic acid#	↓*	-	-	-	-	-	-	-	-
Arachidonic acid	↓*	-	-	↑*	↓*	-	-	-	-
Tryptophan#	-	↓*	-	-	-	-	-	-	-
Cholesterol#	↑*	↑*	↑*	↓*	↓*	↓*	↑*	-	↑*
Ascorbic acid	-	↑*	-	-	-	-	↓*	-	-
TCA#	↓*	↓*	-	↓*	-	-	-	-	-
GCA#	↓*	↓*	-	↓*	-	-	-	-	-
TDCA#	↑*	↑*	↑*	-	↑*	-	-	-	-
CA#	↑*	↓*	-	-	-	-	-	-	-
DCA#	↑*	↑*	↑*	↑*	↑*	-	-	-	-
Muricholic acid	-	-	-	-	-	-	-	-	-

Metabolites	Jejunum	Ileum	Cecum	Colon	Liver	Kidney	Spleen	Lung	Heart
Murocholic acid	-	↓*	↓*	-	-	-	-	-	-
GCDCA#	↑*	-	-	-	-	-	-	-	-
LysoPC(14:0)	-	-	-	-	-	-	-	-	-
LysoPC(15:0)	-	-	-	-	↑*	-	-	↓*	-
LysoPC(16:1)	-	-	-	-	↓*	-	-	-	-
LysoPC(18:0)	↑*	↑*	-	-	-	-	-	-	↑*
LysoPC(18:1)	↑*	-	↑*	↑*	↑*	-	-	-	-
LysoPC(20:1)	-	-	-	-	-	-	-	-	-
LysoPC(20:2)	-	-	-	-	-	-	↑*	-	-
LysoPC(20:4)	-	-	-	-	-	-	-	-	-
LysoPC(P-18:0)	↓*	-	↓*	-	-	↓*	-	-	-
LysoPE(16:0)	-	↓*	↑*	↑*	-	-	-	-	-
LysoPE(16:1)	-	-	-	↑*	-	-	-	↓*	↑*
LysoPE(18:0)	-	-	↑*	↑*	-	-	-	-	-
LysoPE(18:1)	-	↓*	-	↑*	↓*	-	-	-	-
LysoPE(18:2)	↑*	↑*	↑*	↑*	↓*	↓*	↑*	-	-
LysoPE(20:4)	-	↑*	-	-	-	-	-	-	-
LysoPE(22:4)	-	↑*	-	-	-	-	-	-	-
PE(P-16:0e)	↓*	↑*	-	-	-	-	-	-	-

The levels of potential biomarkers labeled with (↓) were down-regulated and (↑) up-regulated in CPT-11 treated rats.

* Mann Whitney U test, $p < 0.05$, in comparison with control group.

# These metabolites were identified by comparing with commercial standards.

Abbreviations: LysoPC, lyso-phosphatidylcholine; LysoPE, lyso-phosphatidylethanolamines; PE, phosphatidylethanolamines; CA: cholic acid; DCA: deoxycholic acid; TCA: taurocholic acid; TDCA: taurodeoxycholic acid; GCA: glycocholic acid; GCDCA: glycochenodeoxycholic acid.
